# Network-level analysis of ageing and its relationship with diseases and tissue regeneration in the mouse liver

**DOI:** 10.1038/s41598-023-31315-2

**Published:** 2023-03-21

**Authors:** Manisri Porukala, P. K. Vinod

**Affiliations:** Centre for Computational Natural Sciences and Bioinformatics, IIIT, Hyderabad, 500032 India

**Keywords:** Cellular signalling networks, Data mining, Gene regulatory networks, Microarrays, Network topology

## Abstract

The liver plays a vital role in maintaining whole-body metabolic homeostasis, compound detoxification and has the unique ability to regenerate itself post-injury. Ageing leads to functional impairment of the liver and predisposes the liver to non-alcoholic fatty liver disease (NAFLD) and hepatocellular carcinoma (HCC). Mapping the molecular changes of the liver with ageing may help to understand the crosstalk of ageing with different liver diseases. A systems-level analysis of the ageing-induced liver changes and its crosstalk with liver-associated conditions is lacking. In the present study, we performed network-level analyses of the ageing liver using mouse transcriptomic data and a protein–protein interaction (PPI) network. A sample-wise analysis using network entropy measure was performed, which showed an increasing trend with ageing and helped to identify ageing genes based on local entropy changes. To gain further insights, we also integrated the differentially expressed genes (DEGs) between young and different age groups with the PPI network and identified core modules and nodes associated with ageing. Finally, we computed the network proximity of the ageing network with different networks of liver diseases and regeneration to quantify the effect of ageing. Our analysis revealed the complex interplay of immune, cancer signalling, and metabolic genes in the ageing liver. We found significant network proximities between ageing and NAFLD, HCC, liver damage conditions, and the early phase of liver regeneration with common nodes including NLRP12, TRP53, GSK3B, CTNNB1, MAT1 and FASN. Overall, our study maps the network-level changes of ageing and their interconnections with the physiology and pathology of the liver.

## Introduction

Ageing is an inevitable complex process altering a multitude of cellular processes. Several studies employing animal models across different organs have outlined the general hallmarks of ageing related to epigenetic modifications, cellular senescence, altered intercellular communication, telomere shortening, nutrient sensing deregulation, mitochondrial dysfunction, stem cell exhaustion, loss of proteostasis, genomic instability, which culminate in the loss of tissue homeostasis^1^. The complexity of ageing process is further heightened by the interconnected feature of some of these processes^2^. Different factors are suggested to cause or contribute to ageing, including DNA damage, free radical (ROS) accumulation and metabolic dysfunction^3^. The oxidative theory of ageing proposes macromolecular damage by the products of metabolism and inefficient repair.

Molecular pathways involving IGF1/GH and mTOR have been implicated in the ageing process^4,5^. Caloric restriction and mTOR inhibition by rapamycin slow down many age-dependent processes and extends lifespan^6,7^. With the advent of high-throughput techniques, biological processes underlying the initiation and progression of ageing can be unfolded at the systems level. However, most studies focused on identifying DEGs and patterns of gene expression in ageing to characterize the transcriptomic changes^8–10^. The upregulation of inflammatory, immune and stress response genes has been reported in different microarray and RNA-Seq experiments of ageing in mice^11,12^. The inflammaging theory postulates ageing accrues inflammation^13^. Tissue-wise transcriptomics study across multiple age groups in mice shows distinct gene expression signatures in different organs, with the liver undergoing extensive changes over time compared to other tissues^9^. The liver is an important metabolic organ that plays a vital role in synthesizing plasma proteins, clotting factors, triglycerides, cholesterol, glycogen, and detoxification^14,15^. Therefore, understanding how ageing rewires the regulatory network of the liver is crucial.

The impairment of structure and function of liver tissue with ageing exacerbates the risk of liver diseases and affects its regeneration potential after damage^16^. Non-alcoholic fatty liver disease (NAFLD) is the commonly seen pathological condition of the liver that evolves into non-alcoholic steatohepatitis (NASH), cirrhosis and hepatocellular carcinoma (HCC). The progression of NAFLD to NASH and further to HCC is favoured by increased inflammation in old age^13^. Interwinding nature of liver ageing and age-related diseases may create a futile cycle of each fuelling the other, leading to a transition from chronological ageing to pathological ageing. In addition to increasing the disease risks, ageing also delays regeneration after partial hepatectomy (PH)^16^. Most of the studies designed to understand liver diseases were dealt independently of each other and without involving the intrinsic process of ageing^17^. Delineating the shared mechanisms inherent to the ageing process and age-related disease shows a road ahead, thereby suggesting therapeutics for liver diseases that are influenced by age.

Network-based approaches can be applied to understand the dynamic changes in gene expression patterns with lifespan and to study the crosstalk between ageing and ageing-related diseases. This provides a systems-level understanding and helps to map dynamical changes. The PPI network provides a scaffold to integrate gene expression data and study the statistical and topological properties of the network in the context of liver ageing and its related diseases. The usage of the PPI network helps to distinguish direct and indirect control compared to the correlation-based co-expression network.

**Figure 1:**
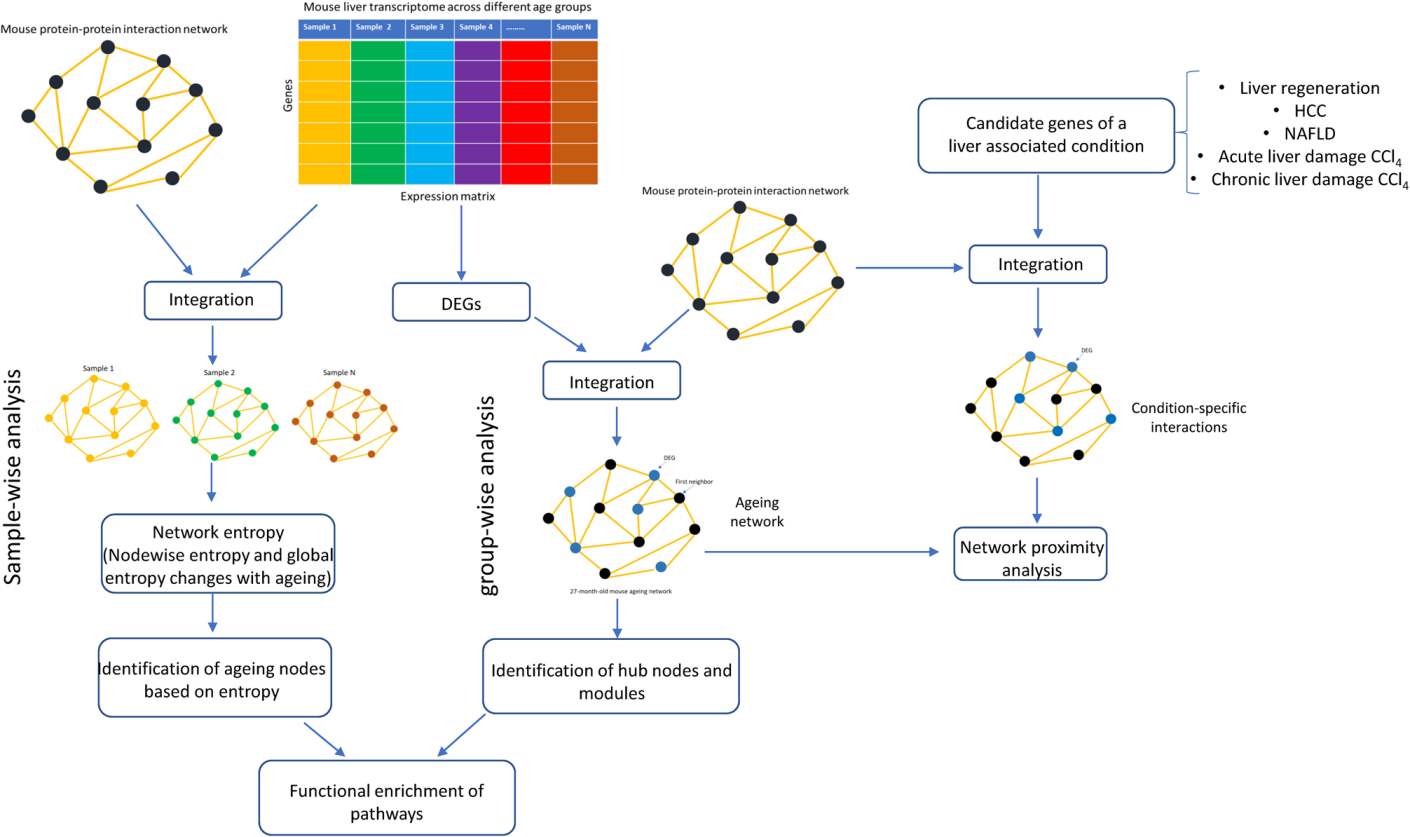
The workflow to study the network-level changes of ageing and its association with tissue regeneration and diseases in the mouse liver.

In this work, we studied how the statistical properties of the liver network change with ageing by integrating the PPI network and mRNA expression profiles of mouse liver samples across ten different age groups available from Tabula Muris Consortium^10^. Network entropy quantifying randomness offers a new perspective for studying ageing and diseases. We show that entropy of the liver network increases with ageing, indicating the increase in randomness due to network disruption by genomic alterations. We computed the local entropy measure to identify genes and pathways associated with ageing. The genomic alterations in ageing may either increase or decrease the randomness of the local connectivity patterns (change the probability of interactions)^18–20^. A decrease in entropy signifies specific signaling interactions with higher weights, while an increase in entropy signifies the unpredictable nature of interactions. To gain further insights, we integrated the DEGs between young and different age groups with the PPI network to identify core modules and nodes that show changes in local and global topological network measures with ageing. Finally, we computed the network proximity of the ageing network with different networks of liver diseases and regeneration to study the effect of ageing. The workflow of the study is shown in Fig. 1.

## Methods

### Network entropy-based approach to analyze liver ageing:

Transcriptomics data (bulk RNA-Seq) of mouse liver tissue with age groups 3, 6, 9, 12, 15, 18, 21, 24, and 27 months was obtained from GEO with accession number GSE132040 (Tabula Muris Consortium). Each age group has 3–4 replicate samples. The raw count data was normalized using variance stabilizing transformation (VST)^21^. An entropy-based approach was used to integrate the gene expression data with the PPI network. Mouse-specific STRING PPI network (10,596 nodes and 86,074 edges) with interaction confidence-score cut-off ≥ 0.9 was used as the initial PPI network. A network characterised by a specific number of nodes, edges and edge weights is considered an instance in an ensemble of large number of networks with similar features. This system has two sets of observables related to degree sequence and distribution of edge weights. The entropy metric of a network is given by calculating the maximum entropy of the ensemble satisfying the given constraints (with the identical topological and spatial structure of the network) rather than the original network (see supplementary methods)^20^. For integration of gene expression and PPI network, nodes in the PPI network are assigned with their corresponding gene expression values specific to a particular sample. The edges connecting nodes are weighted as the distance between gene expression values. The edge weights are converted to a distribution by partitioning them into number of bins equal to the square root of number of nodes in the network. While building the network, nodes with zero gene expression value are removed from the network. Hence, the final network which is subjected to entropy maximization differs from the original PPI network and is sample-specific. Therefore, the static PPI network evolves when it is integrated with sample-specific gene expression.

Further, the network entropy of a sample can be used to derive the entropy associated with a single node that takes the form of Shannon entropy (local entropy) (see Supplementary Methods). The Wilcoxon rank sum test was applied to identify nodes showing significant differences (FDR < 0.05) in entropy between groups of samples at the single-node level. This analysis was performed by considering samples of 3–6 months old mice as the younger age group and samples of 24–27 months old mice as the older age group. The pathway enrichment analysis of nodes that display significant differences in single-node was performed using Enrichr^22^ to obtain significantly affected pathways (adjusted p-value < 0.05).

### Graph theoretical analysis of ageing PPI network

Unlike the previous approach, which integrates sample-specific gene expression with the PPI network for entropy calculation, we alternatively constructed the age group-wise networks to compare the local and global network measure changes with ageing. For this, DEGs comparing 3 months old mice with 18, 24 and 27 months old were used for building individual PPI networks. DEGs identified using the DESeq2 pipeline were integrated with STRING PPI (confidence-score cut-off ≥ 0.9) to build individual networks for comparison. Each PPI network was further expanded by including the first neighbours of DEGs, and this network was considered for all the downstream analyses.

Each PPI network was analyzed using the CytoHubba plugin in Cytoscape 3.9.0^23^. A PPI network is assumed to be an undirected network *G* = (*V, E*) with *V* as set of nodes and *E* as set of edges connecting the nodes. CytoHubba identifies essential hub nodes and subnetworks within the PPI network using various local and global metrics^24^. Each of these metrics is associated with a function *F* which assigns every node *v* a numeric value *F*(*v*)*.* A node *u* is awarded a higher rank compared to another node *v* if *F*(*u*) > *F*(*v*). A local ranking method only considers the relationship between the node and its direct neighbours to calculate the score. On the other hand, a global ranking method assigns a score to a node based on its relationship with the entire network.

For local measure, we used Maximal Clique Centrality (MCC), which is based on the concept of a clique that emphasizes the highly connected clusters within a network. A clique *C* in a network is a subset of nodes (*C *⊆* V*) such that every pair of nodes is connected. Further, if such a clique cannot be extended by adding one or more other nodes (for any *x* ∈ *V\C, C* ∪ {*x*} is not a clique), it becomes a maximal clique. MCC score for a node *v* is given as

$$MCC\left(v\right)= \sum_{C\,\epsilon\,S(v)}\left(\left|C\right|-1\right) !$$

where *S*(*v*) is the collection of maximal cliques *C* which contains *v*, and (|*C*| − 1)! is the product of all positive integers less than |*C*|. Therefore, a node with a higher MCC score implies that it is part of larger cliques or many smaller cliques or both.

In addition to the connectivity of a node, its spatial position in the network also influences the communication among other nodes. To capture the nodes that regulate the information flow within the network, we used two shortest path-based global measures, Bottleneck centrality (BN) and Betweenness centrality (BW), for each node. Bottlenecks are considered to act as bridges holding crucial functional and dynamic properties in the network^25^. While the *BN*(*v*) score of node *v* is based on the shortest path trees of all other nodes in the network, *BW*(*v*) is based on the number of shortest paths between every pair of nodes traversing the node *v*. Scoring of *BN*(*v*) for a node *v* begins with the construction of tree *T*_*s*_ of shortest paths from a node *s* to all other nodes in the network, followed by enumeration of the number of these shortest paths going through node *v.* This process is iterated for all *s* ∈ *V.* A node *v* in the shortest path tree *T*_*s*_ is considered as a bottleneck if more than $\frac{|V\left({T}_{s}\right)|}{4}$ of the paths in the tree cross it^26^, where |*V*(*T*_*s*_)| is the number of nodes in the tree. Finally, *BN*(*v*) of node *v* is scored as the number of such shortest path trees where it is considered as a bottleneck and is given by

$$BN\left(v\right)= \sum_{s\,\epsilon\,V}{p}_{s}(v),$$

where $${p}_{s}\left(v\right)=\left\{\begin{array}{c}1, if\,number\,of\,paths\,from\,s\,to\,V\left({T}_{s}\right)\backslash\,v\,through v>\frac{|V\left({T}_{s}\right)|}{4}\\ 0, otherwise\end{array}\right..$$  

Betweenness centrality *BW*(*v*) of node *v* in the connected component *C*(*v*) containing *v* is the sum of fraction of shortest paths between every pair of nodes *s* and *t* traversing through *v, σ*_*st*_(*v*), to the total number of shortest paths between every pair of nodes *s* and *t,* σ_*st,*_ and is given by

$$BW\left(v\right)= {\sum }_{s \ne t \ne v \epsilon C(v)}\frac{{\sigma }_{st}(v)}{{\sigma }_{st}}.$$

Further, the densely connected components of the network that are likely to form molecular complexes were identified using the Molecular Complex Detection (MCODE)^27^ program’s default settings in Cytoscape. MCODE clusters with scores ≥ 5 were further analysed by using Enrichr^22^ for pathway enrichment.

### Network-based proximity analysis

Gaining insights into the interconnectedness of disease genes with ageing within the PPI network helps to understand the risk of ageing. If disease modules in an interactome overlap or are significantly closer to ageing modules, then perturbations due to ageing may affect pathways in disease or drive its progression. Proximity analysis was performed to study the associations between the ageing liver and each of the perturbed liver conditions (liver regeneration post-PH, NAFLD, HCC, acute liver damage by CCl_4_ and chronic liver damage by CCl_4_)_._ The association between two conditions was quantified using a network proximity metric^28^:

$${<d}_{AB}^{C}>=\frac{1}{||A||+||B||}\left(\sum_{a\in A}{min}_{b\in B}d(a,b) + \sum_{b\in B}{min}_{a\in A}d(a,b)\right),$$

where *d*(*a*,* b*) represents the shortest path length between gene *a* from condition A and gene *b* from condition B in the interactome.

The significance of this distance metric was evaluated using the Z-score of permutation test by randomly selecting nodes from the whole network with degree distributions similar to that of the nodes in the two sets. Z-scores were calculated by permutation tests of 1,000 repetitions as follows:

$${Z}_{{d}_{AB}} = \frac{{d}_{AB }- {d}_{m} }{{\sigma }_{m}},$$

where *d*_*m*_ and *σ*_*m*_ are the mean and standard deviation of the permutation test.

**Figure 2:**
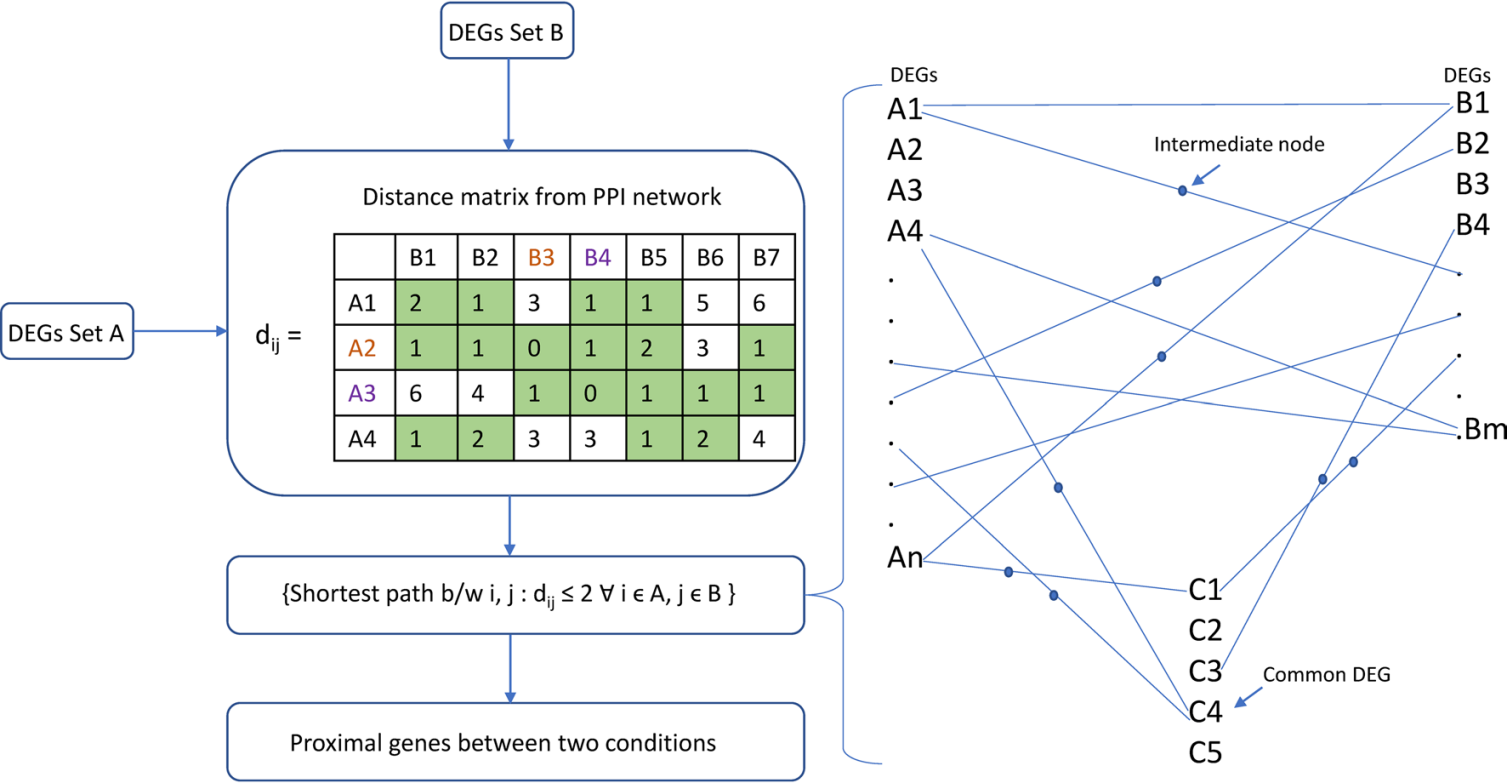
The flowchart to identify proximity genes between two conditions A and B. The shortest path connecting each pair of genes was identified. The nodes (A1, A2…An and B1, B2…Bm) can be directly connected or through an intermediate node. C represents common nodes between two conditions.

Candidate gene lists for ageing and other conditions were selected from different studies with similar mouse strains (Table 1). DEGs between 3 and 27 months old mice were considered as signatures of ageing for proximity analysis. We also performed proximity analysis using DEGs from different age groups, including 12, 18 and 21 months, for comparison. Candidates for different phases of liver regeneration were considered by taking the union of DEGs of early-phase (1, 4, 10 h post-PH compared to pre-PH), mid-phase (36, 44, 48 h post-PH compared to pre-PH), and late-phase (1- and 4-weeks post-PH compared to pre-PH). We also included DEGs of sham-operated control samples at different phases, i.e., early-phase (1, 4, 10 h post sham surgery) and mid-phase (48 h post sham surgery). Candidate genes for NAFLD and HCC (DEGs between healthy control and disease) were pooled from their respective studies (Table 1). The proximity analysis was performed using the high confidence mouse-specific STRING PPI network (confidence score ≥ 0.9). Two conditions with Z-score <  − 1.5 and FDR < 0.05 were considered significantly proximal. To infer the biological significance of proximity of ageing signatures with other conditions, the shortest path connecting each pair of DEG sets was identified as depicted in Fig. 2.

**Table 1:** Datasets used to define the list of candidate genes for different liver associated conditions.

Liver condition	Accession no.	Experimental mouse model	Age of mice during sample collection	Strain
Aging	GSE132040	Age group spanning 3–27 months	3, 6, 9, 12, 15, 18, 21, 24, 27 months old	C57/BL6J
Regeneration and sham-operated control	GSE95135	12–14 weeks old mice	3 months old	C57/BL6J
NAFLD	GSE148080	Normal diet beginning at 8 weeks followed by 8–16 weeks of normal diet/high sucrose diet	8 months old	C57/BL6J
	GSE184019	Normal diet at 8 weeks followed by 3 weeks of normal diet/high sucrose diet. Samples collected at 11 weeks	3 months old	C57/BL6J
HCC	GSE132728	Single dose of DEN at 2 weeks followed by weekly twice dose of CCl_4_ from 8 to 24 weeks	6 months old	C57/BL6J
	GSE89689	Single dose of DEN at 2 weeks followed by first dose of CCl_4_ dose 4 weeks later. Further weekly dose of CCL_4_ for 15 weeks. Final samples were collected 10 weeks after the last dose of CCl_4_	8 months old	C57/BL6J
Acute damage (CCl_4_)	GSE167033	8–10 weeks old mice were administered with CCl_4._ Samples were collected 2 and 8 h post treatment, 1, 2, 4, 8, 16 days post treatment	2–3 months old	C57/BL6/N
Chronic damage (CCl_4_)	GSE167216	8–10 weeks old mice were treated with CCl_4_ twice a week for 2, 6, 12 months	4, 8, 12 months old	C57/BL6/N

## Results

### Alteration in network entropy with ageing in the mouse liver

**Figure 3:**
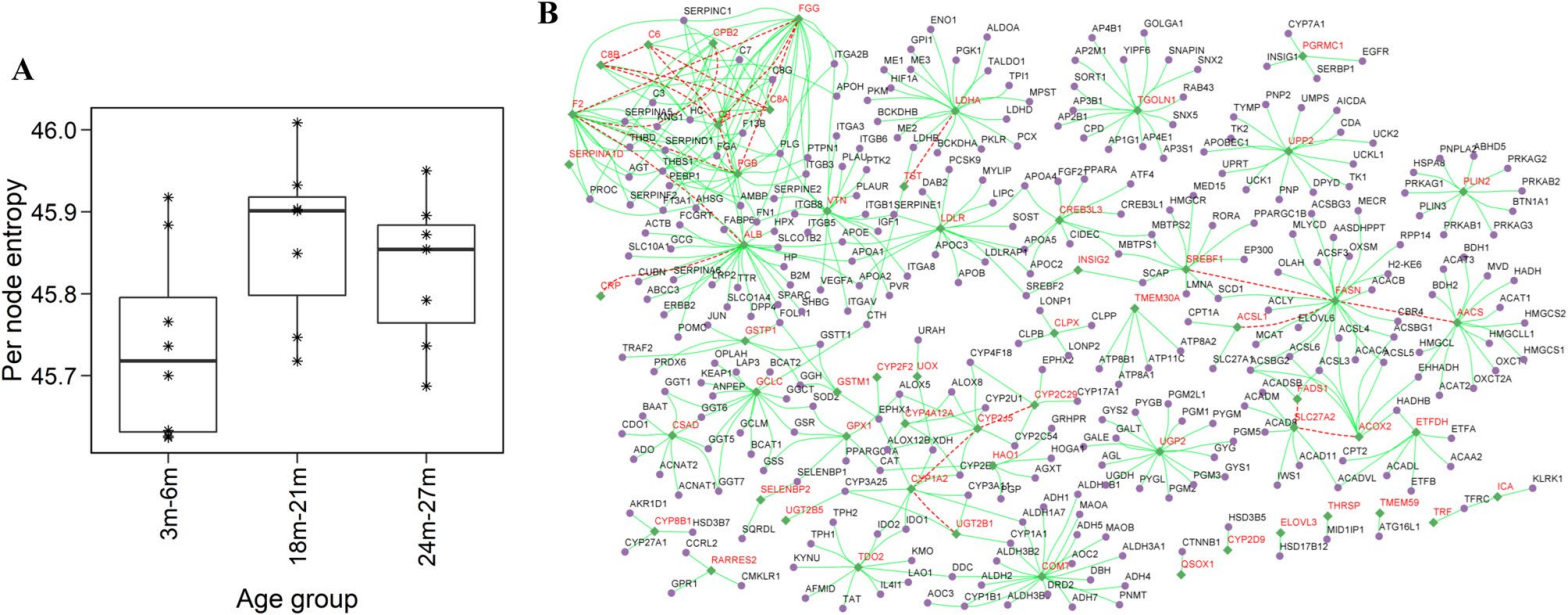
Network entropy-based analysis of liver ageing network. (**A**) Boxplot showing the change in network entropy across different age groups. Sample-wise entropy is calculated and is normalized by number of nodes in its corresponding network. (**B**) Network of top 50 nodes with significant change in local entropy and their neighbours. Top nodes are colored in red and the edges connecting them are shown with red dashed lines. Edges between neighbours are not shown.

We used the network entropy measure to study ageing. The sample-wise gene expression was integrated with the PPI network. The estimation of network entropy from the liver gene expression data shows that entropy increases in old age (18–21 months) compared to the young (3–6 months) mice (Fig. 3A). This is in agreement with other studies that used a similar approach to study the progression of ageing in the context of skeletal muscle and T-lymphocytes^20,29^. The 18–21 months is a tipping point after which entropy slightly decreases in the oldest age group (24–27 months). This reveals that the liver tissue undergoes network disorganization with ageing, increasing the disorderness or randomness. We also performed local differential entropy analysis between young and old age groups to identify nodes showing significant increase in randomness. We identified 684 nodes with significantly differing single node entropies (Wilcoxon Rank sum test q-value < 0.05, absolute difference in median > 0.03) between young (3m–6m age) and oldest age (24m–27m) groups. The pathway enrichment of these genes revealed that Complement and coagulation cascades, Cytokine–cytokine receptor interaction, Xenobiotics metabolism, steroid hormone biosynthesis, NFKβ signalling pathway, PI3-AKT signalling pathway, and MAPK signalling are significantly affected (Table 2). The entropy-based approach captures relevant pathways associated with ageing.

**Table 2:** Pathway enrichment of nodes that showing significant change in entropy (FDR < 0.05) between young (3–6 months) and old (24–27 months) mice with absolute mean difference greater than 0.03.

S. no.	KEGG pathway	Overlap	p-value	Adj p-value
1	Complement and coagulation cascades	18/88	8.46E−10	2.42E−07
2	Cytokine–cytokine receptor interaction	30/292	8.91E−08	1.27E−05
3	Primary immunodeficiency	10/36	2.33E−07	2.15E−05
4	Metabolism of xenobiotics by cytochrome P450	13/66	3.01E−07	2.15E−05
5	Chemical carcinogenesis	15/94	6.66E−07	3.81E−05
6	Steroid hormone biosynthesis	14/89	1.87E−06	8.88E−05
7	PI3K-Akt signaling pathway	31/357	2.17E−06	8.88E−05
8	Pathways in cancer	39/535	8.15E−06	0.000291
9	Pentose and glucuronate interconversions	8/34	1.48E−05	0.000471
10	MAPK signaling pathway	25/294	2.93E−05	0.000796
11	Cholinergic synapse	14/113	3.16E−05	0.000796
12	Drug metabolism	14/114	3.49E−05	0.000796
13	Th1 and Th2 cell differentiation	12/87	3.94E−05	0.000796
14	T cell receptor signaling pathway	13/101	4.01E−05	0.000796
15	Fatty acid degradation	9/50	4.33E−05	0.000796

The top-ranking nodes based on increase in entropy belong to the cytochrome P450 superfamily (CYP7B1, CYP2D9, CYP2F2, CYP2C29**)** and UDP-glucuronosyltransferases (UGT2B5, UGT2B36 and UGT2B1), which are linked to drug metabolism and steroid hormone synthesis (Fig. 3B). The entropy increase is observed with FGG, FGB and VTN, which are associated with ECM and wound healing. VTN encodes for a secreted protein vitronectin that inhibits the membrane-damaging effect of the terminal cytolytic complement pathway (endothelial cells)^30^. TDO2 shows an increase in entropy and is linked to changes in tryptophan and kynurenine (Kyn). Tryptophan metabolism controls the inflammation-associated decline in age-related tissue homeostasis (inflammaging)^31^.

Fatty acid oxidation genes ACSL1, ACADVL, ETFDH, ACOX2, HADHA, HSD17B4 and fatty acid transport gene SLC27A2 show an increase in entropy. The involvement of mitochondrial and peroxisome genes linked to fatty acid oxidation suggests an interplay between peroxisome-mitochondria in liver ageing^32^. CREB3L3, which cooperates with PPARA to regulate the expression of genes involved in fatty acid metabolism, also shows an increase in entropy (Fig. 3B). On the other hand, the entropy of lipid synthesis genes FASN, SREBF1, FADS1 and AACS and lipid transport gene LDLR decrease with ageing. Interestingly, the entropy of PGRMC1 and INSIG2 that regulate hepatic de novo lipogenesis via SREBF1 increases. Similarly, PLIN2, a gene associated with the metabolism of intracellular lipid droplets (LDs), also shows an increase with ageing.

Further, genes of glutathione metabolism show a change in entropy with ageing. Glutathione S-transferase (GSTs) GSTP1 shows an increase in entropy, while GSTM1 shows a decrease in entropy. GSTs are the Phase-II enzymes that protect the cells against damage induced by electrophiles and products of oxidative stress. They are shown to have anti-ageing effect^33^. GPTX1, which catalyzes the reduction of hydrogen peroxide (H_2_O_2_) by GSH, also shows an increase in entropy along with GCLC, an essential gene for GSH synthesis. RARRES2, which encodes a chemoattractant protein (Chemerin) secreted by the liver, shows a decrease in entropy with ageing. Chemerin is a modulator of immune response by promoting the chemotaxis of numerous immune cell types and it has a role in pathophysiological conditions including HCC and NAFLD^34^.

The overlap of entropy-based genes with DEGs between 3- and 27-months old mice shows only a few overlaps indicating that genes identified based on statistical properties of the underlying network are unique (Supplementary Fig. S1A). We also compared the entropy-based candidate genes with the curated mouse immune genes^35^ (Supplementary Fig. S1B). The entropy-based analysis also identifies distinct immune-ageing genes compared to DEG analysis with a small overlap. This suggests that ageing is characterized by global changes in the immune system. Non-overlapping 454 genes also include genes related to neurodegeneration (DNAHs) and protein digestion and absorption (Collagens). Immune markers unique to entropy-based analysis include genes VTN, FGB and FGG.

### Core gene expression modules associated with ageing

We also alternatively explored the ageing gene expression changes at the network level by integrating DEGs and PPI network. We expanded the network to include the first neighbours of DEGs. The PPI network built from DEGs comparing extreme age groups (3 and 27 months) and their first neighbours resulted in 38,764 edges connecting 3770 nodes. Similarly, we also constructed an ageing network for other age groups (18, 21 and 24 months) for comparative analysis. We first clustered genes based on network topology to identify densely connected regions using MCODE.

**Figure 4:**
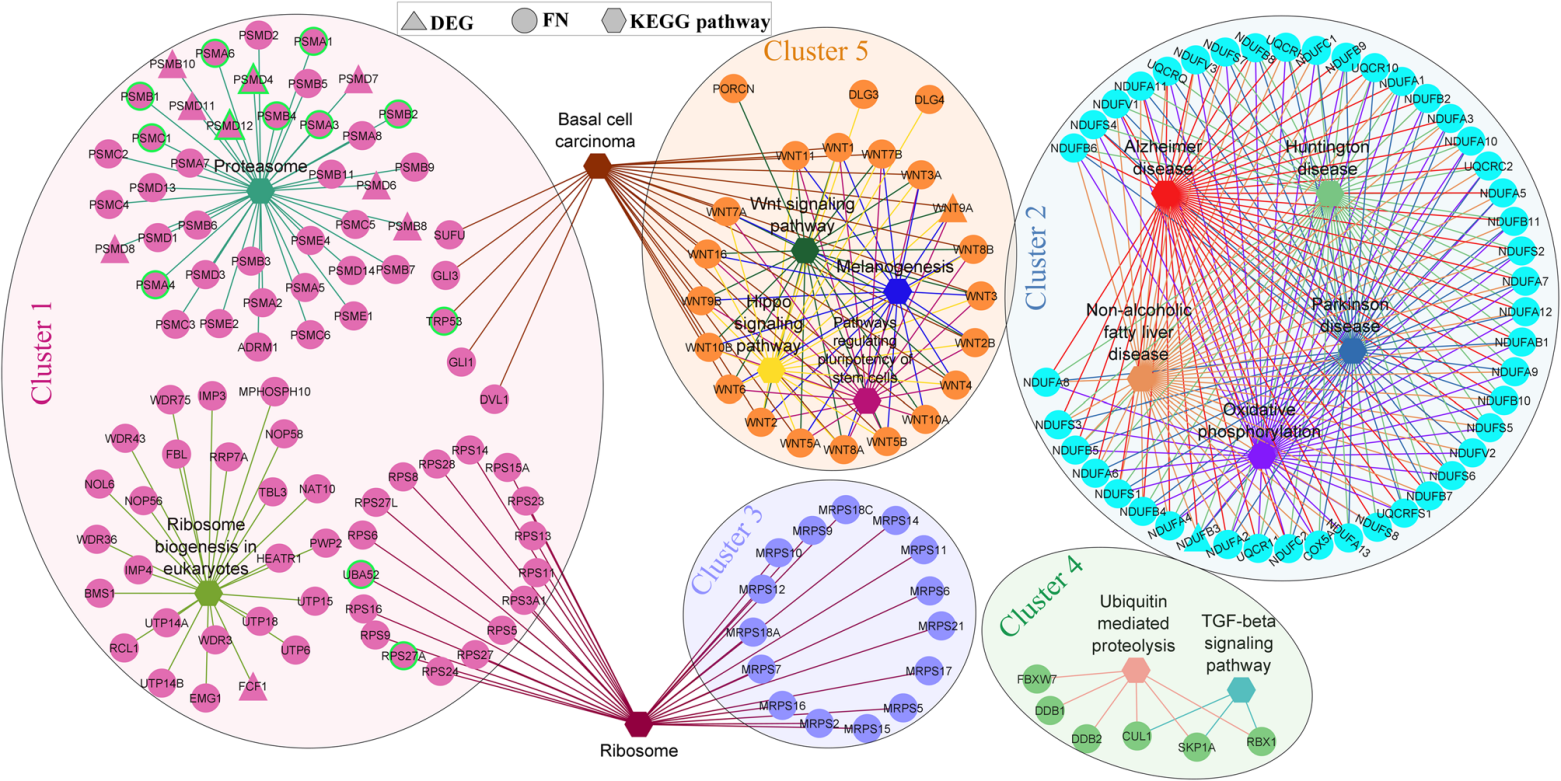
Top five MCODE clusters of the ageing network obtained using the DEGs between 3 and 27 month old mice and their first neighbours. Pathway enrichment of clusters are shown along with the gene information. Hub nodes/genes are highlighted with green border.

The modular analysis of the liver ageing network (3 and 27 months) shows that genes corresponding to pathways such as Ribosome, Proteasome and Oxidative phosphorylation are associated with top-scoring clusters (Fig. 4). These pathways are also found in the 18- and 24-months old mice ageing network (Supplementary Tables S1 and S2). Signalling pathways (mainly Wnts) regulating pluripotency of stem cells emerged as a significant pathway in the oldest 27-month age group. The clusters from 18 to 24-month networks are also associated with cell cycle, DNA repair, p53 signalling pathway and senescence. The enrichment of top clusters shows the relationship to NAFLD, basal cell carcinoma, neurodegenerative diseases, and viral infection.

We also identified critical nodes based on local and global network measures. Topological analysis of the ageing network based on local (MCC) and global (Bottleneck and Betweenness) metrics show that RPS27A and TRP53 are critical nodes in the network (Table 3). Other nodes of global importance in the network include AKT1, SRC, CTNNB1, and EGFR, while genes associated with proteosome (PSMB2, PSMA6, PSMB4, PSMA1, PSMB1, PSMA3, PSMD12, PSMC1, PSMD3, PSMA4, PSMD4) are locally important. It is also observed that RPS27A and TRP53 are not only the nodes of global and local importance nodes in the extreme age group but also form the early signs of ageing (Table 3).

**Table 3:** Hub nodes of liver ageing network based on local and global network measures.

Network measure	3 vs 18 months	3 vs 24 months	3 vs 27 months
MCC	NDUFB7, NDUFB9, NDUFAB1, NDUFB8, NDUFA5, NDUFA6, NDUFV2, NDUFB10, NDUFA12, NDUFB5, NDUFA8, NDUFS8, NDUFS7, NDUFA9, NDUFA10, NDUFV1, NDUFS1, NDUFS3, UQCRFS1, NDUFS2, UQCRC1, UQCRC2, COPS3, COPS4, COPS2, COPS5	PSMD1, PSMC3, PSMC6, PSMD11, PSMC5, PSMD12, PSMC1, PSMD3, PSMB7, PSMA5, PSMB5, PSMA2, PSMD6, PSMA1, PSMB3, PSMB2, PSMB10, PSMA3, PSMA6, PSMA4, PSMA7, PSMB4, PSMB6, PSMB1, PSMD4, PSMB8, PSMA8, PSMB9, CDC6 RELA, CCND1, UBA52, UBC, UBB, RPS27A, TRP53, CCNB1, CDK1	PSMB2, PSMA6, PSMB4, PSMA1, PSMA3, PSMD12, PSMC1, PSMD3, PSMA4, PSMD4, PTEN, RELA, UBB, UBC, UBA52, RPS27A, CDK1, TRP53
Bottleneck	AKT1, SRC, EGFR, CTNNB1, TRP53, RAC1, JUN	TRP53, ESR1, AKT1, CTNNB1	PTEN, UBA52, RPS27A, TRP53, AKT1, SRC, CTNNB1, ESR1
Betweenness	AKT1, SRC, EGFR, CTNNB1, TRP53, ESR1, RAC1	RPS27A, TRP53, ESR1, AKT1, CTNNB1, KRAS, SRC, RHOA	RPS27A, TRP53, AKT1, SRC, CTNNB1, EGFR, ESR1

### Relationship between ageing and pathways associated with liver diseases and regeneration

**Figure 5:**
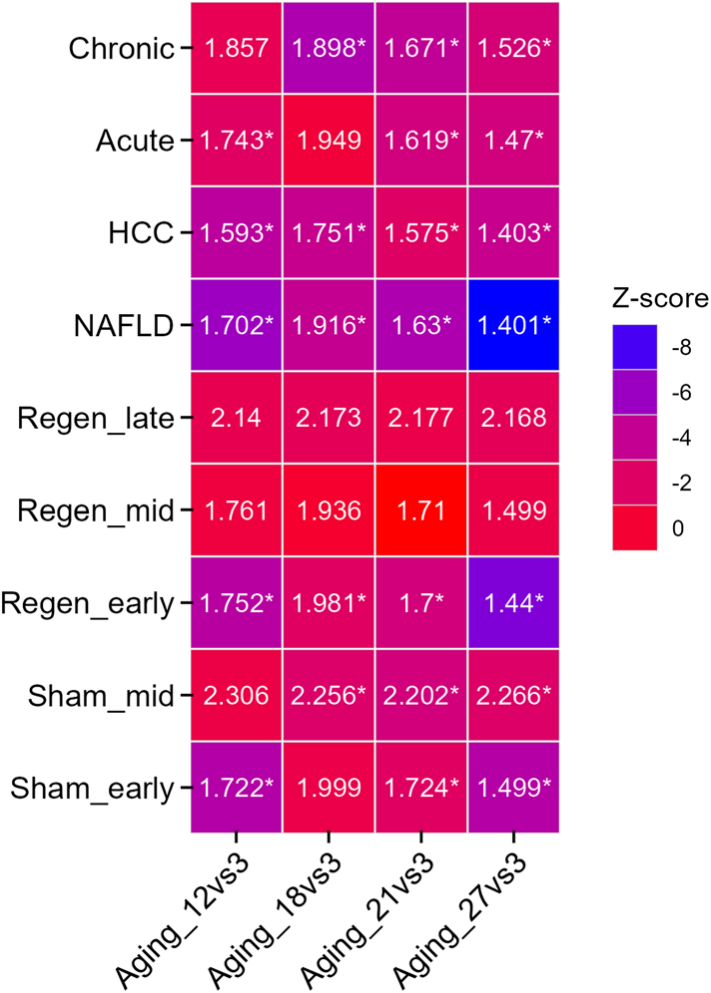
Network proximity of ageing with different liver-associated conditions: early (Regen_early), mid (Regen_mid) and late (Regen_late) phases of liver regeneration, early and mid-phases of sham-operated control, NAFLD, HCC, Acute and chronic liver damages. The proximity is explored for different age groups (12, 18, 21 and 27 months). Text in the tiles represents proximity distance. *Indicates FDR < 0.05 and Z-score < − 1.5.

Ageing can increase the susceptibility to liver diseases like HCC and NAFLD and affect the ability of the liver to regenerate after damage. We hypothesized that this might arise due to shared or related pathways associated with liver diseases and regeneration. We performed network proximity analysis using condition-specific DEGs to study the relationship between ageing and perturbations that influence liver function. The network distance was quantified using the mouse PPI network. We found significant proximities between ageing and liver-related pathologies such as NAFLD, HCC and acute and chronic damage by CCl_4_ by integrating DEGs and mouse PPI network (Fig. 5). The proximity distance decreases with an increase in the age of mice.

Proximity analysis between ageing and the early phase of liver regeneration (1, 4, and 10 h post-PH) shows that older age groups are significantly proximal to the liver regeneration module. This proximity may influence the liver regeneration process in ageing. Ageing is shown to delay liver regeneration post-PH. However, the mid and late phases of liver regeneration associated with cell cycle and termination phases, respectively, are not significantly proximal to the ageing module (Fig. 5). Therefore, proximity analysis captures and quantifies the impact imposed by ageing on regeneration at the network level. The early phase of sham-operated control is also proximal to the older age compared to the young age. This is consistent with the observation that the early phase of sham-operated control and liver regeneration is similar^36^. Further, the proximity of the mid-phase of sham-operated control to the ageing network increases compared to the early phase.

**Figure 6:**
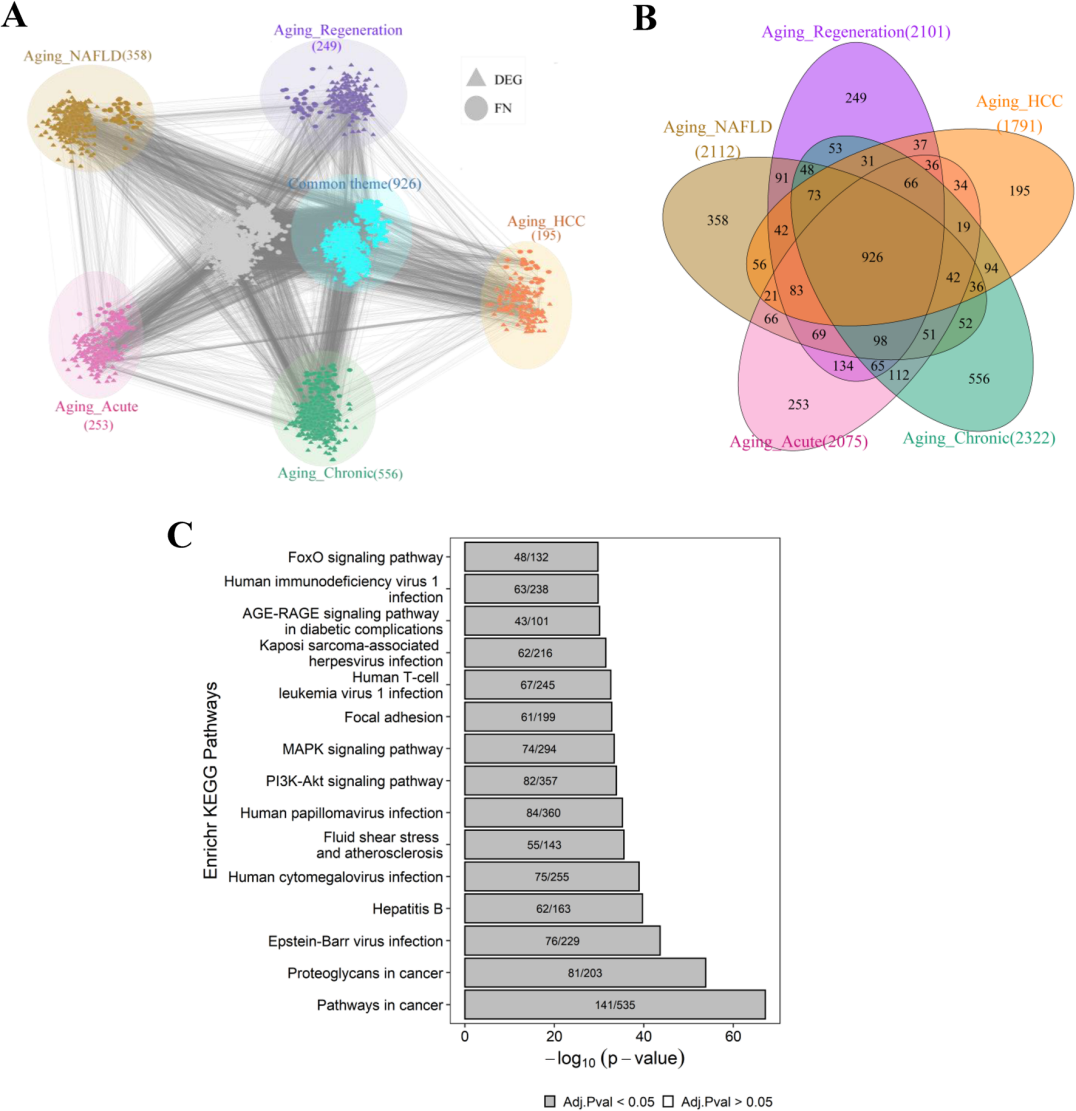
Overlap of proximity nodes obtained in the pairwise comparison of ageing and different liver associated conditions. (**A**) Crosstalk (interactions) between nodes of different liver-associated conditions are shown using the PPI network. The common theme comprises of nodes that are present in all comparisons. Nodes that are neither part of common theme nor specific to a condition are shown in grey. A node that is a DEG in at least one condition is shown by triangle and first neighbour (FN) of DEG is shown by circle. (**B**) Venn diagram showing the number of nodes overlapping between different pairwise comparisons. (**C**) The pathway enrichment of 926 genes in the common theme is shown.

**Figure 7:**
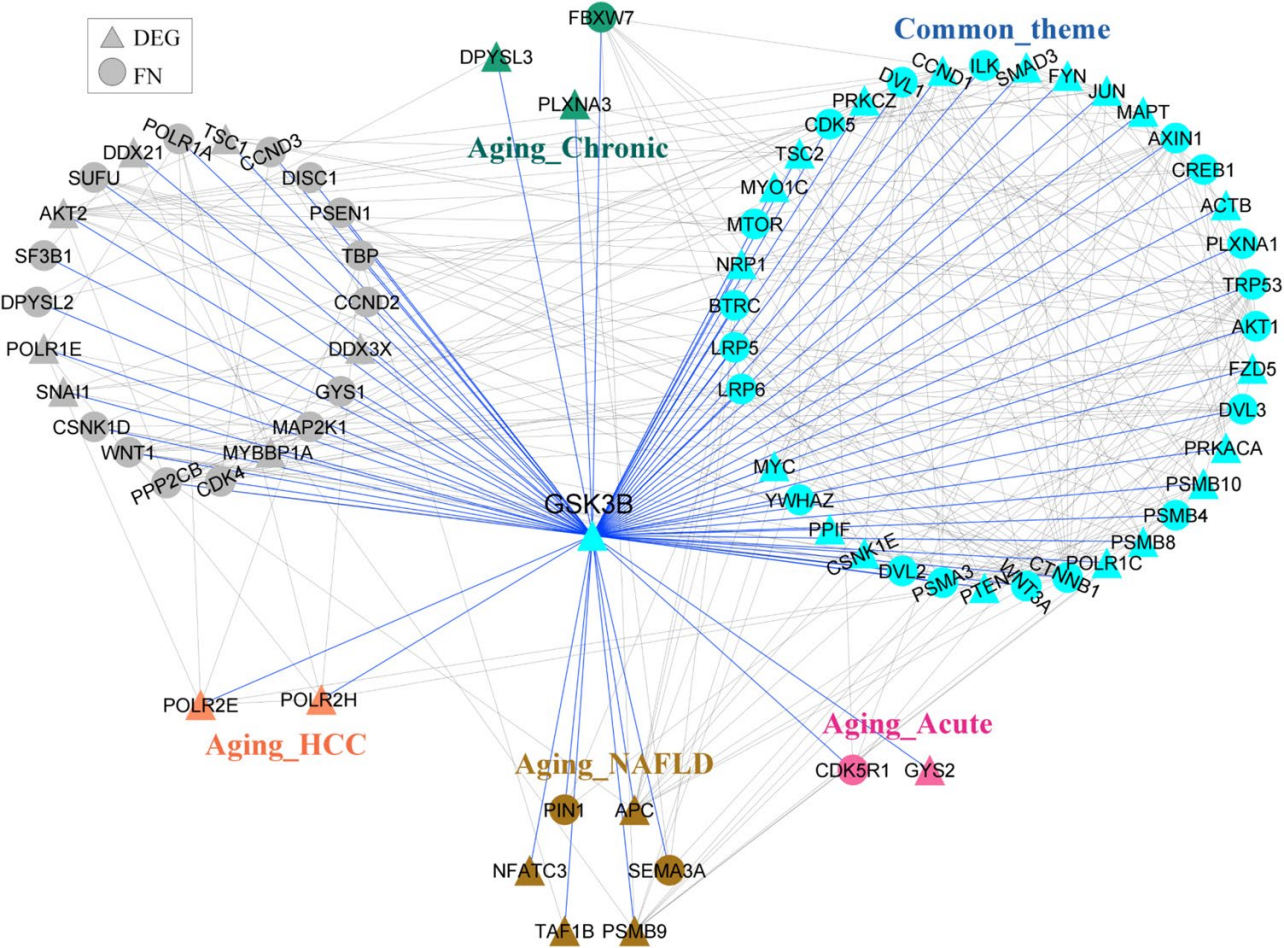
Interacting partners of candidate gene, GSK3β, present in the common theme.

**Figure 8:**
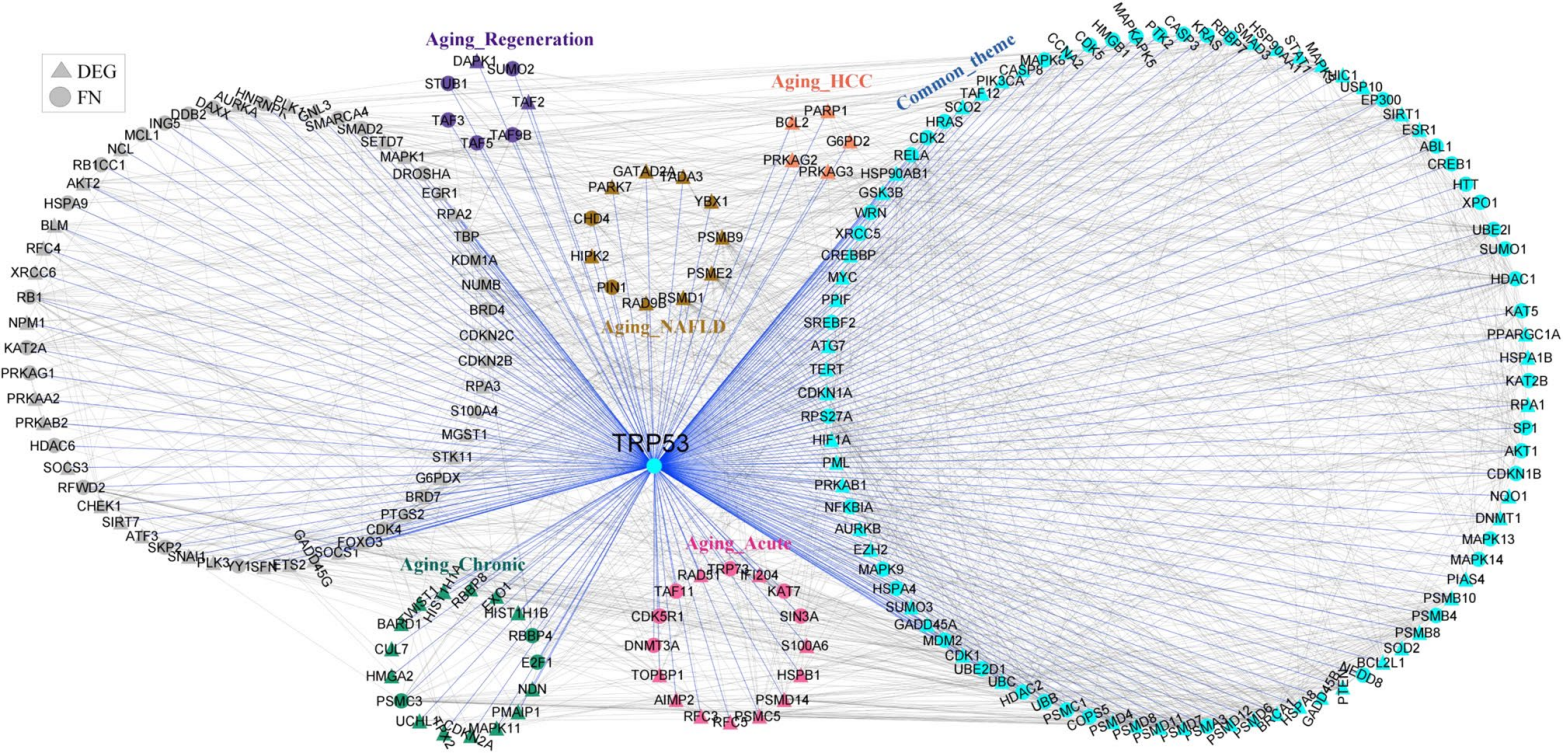
Interacting partners of candidate gene, TRP53, present in the common theme.

To probe the qualitative picture of proximity analysis, we identified nodes falling in the shortest path between every ageing gene and all candidate genes of other conditions (Fig. 2). This resulted in 2101, 2112, 1791, 2075 and 2322 nodes in the pairwise comparisons: ageing and regeneration, ageing and NAFLD, ageing and HCC, ageing and acute damage, ageing and chronic damage, respectively. Nodes from each comparison were collectively projected onto the PPI network (Fig. 6A). The connectivity pattern suggests that ageing is connected to different conditions through intermediate nodes between condition-specific DEGs. We observed a common theme of 926 proximal molecular players connecting ageing with different liver conditions emerges (Fig. 6B). This converges on important KEGG pathways such as pathways in cancer, proteoglycans in cancer, Epstein-Barr virus infection, PI3K-Akt signalling pathway and MAPK signalling pathway (Fig. 6C). GRB2, SOS, RAS, RAF and ERK1/2, are the important molecular players present in the top pathways associated with the common theme. GSK3B is another interesting candidate gene common across ageing, NAFLD and HCC (Fig. 7). It is upregulated in NAFLD and downregulated in HCC. GSK3B connects different conditions via CTNNB1. TRP53 signalling pathway also connects ageing to liver-associated conditions. This may control cell cycle entry by regulating genes such as CCND1, CDKN1A and GADD45A (Fig. 8). Both GSK3B and TRP53 interaction is also part of the common theme. The overlap of 926 genes with curated mouse-specific immune genes shows that 366 genes are common (Supplementary Fig. S2), with NFKβ as a key transcriptional factor. NFKβ regulates innate and adaptive immunity and is the master regulator of inflammatory responses^37^. We also identified NLRP12 as a common candidate gene that plays the role of a mitigator of inflammation. It is upregulated in the early phase of liver regeneration while downregulated in ageing, NAFLD, and acute and chronic liver damage.

In addition to the immune system, we also explored other common relationships between ageing and different liver conditions. Lipid (FASN, HMGCR, SREBF1) and bile acid synthesis (CYP27A1) genes are differentially expressed in ageing and liver regeneration. FASN, HMGCR and SREBF1 are upregulated in ageing. Mitochondrial fatty acid β-oxidation (FADS1, HADHA, HADHB, ACSL1, ACADVL, CPT2, ECI2) also shows this differential pattern. CREB3L3 is differentially expressed across conditions. It is upregulated in liver regeneration and downregulated in ageing. PCSK9, which plays a role in cholesterol homeostasis, is downregulated in the early phase of regeneration and upregulated in ageing and NAFLD. It protects the liver against steatosis and liver injury. On the other hand, ANGPTL4, which facilitates the accumulation of TAG by inhibiting LPL, is downregulated in ageing while it is upregulated in liver regeneration.

Ageing also influences amino acid metabolism. Genes of one carbon metabolism (DHFR, MTHFD1, BHMT, SHMT1/2, MAT1A, MTR, TYMS) are affected across conditions. *S*-adenosyl-methionine (SAM) metabolism controlled by MAT1 is significantly upregulated in liver regeneration compared to ageing and is downregulated in HCC. MAT1 regulates the production of SAM from methionine, which is required for methylation reactions inside the cell. In NAD metabolism, NAMPT is upregulated in ageing, while NNMT is upregulated in regeneration and downregulated in NAFLD and HCC. Genes involved in BCAA catabolism, glutamine catabolism (GLS2), aspartate synthesis (ASS1), and Tryptophan metabolism (TDO2) are also affected across different liver conditions.

## Discussion

Ageing can lead to functional impairment of liver and predisposes the liver to NAFLD and HCC. The liver has a unique ability to regenerate itself post-injury and help in whole-body metabolic homeostasis and compound detoxification. Mapping the molecular changes of the liver with ageing may help to understand how ageing influences liver function and predisposes the liver to different pathological conditions. A systems-level analysis of the ageing-induced liver changes and its crosstalk with the pathology of liver diseases is lacking. In the present study, we performed a network-level analysis of liver ageing using transcriptomic data of ageing and the PPI network. We used network entropy measure to identify nodes and pathways that show significant entropy changes in ageing. Further, we also performed the topological analysis of the ageing network by considering the nodes differentially expressed in ageing and their first neighbours to identify the core modules of ageing. This framework was also used to study the proximity of the ageing network with liver regeneration and disease networks. We showed proximity measure provides insights into the interconnection between ageing and liver-associated conditions.

We observed an increase in entropy with ageing liver with the subtle difference between old and oldest groups (Fig. 3). The entropy-based approach captured the relevant pathway-level changes linked to ageing and helped identify novel candidate genes. The entropy change is driven by the selected group of genes belonging to the immune, complement and coagulation cascade, lipid metabolism, cytochrome P450 and UDP-glucuronosyltransferases. Immune and lipid metabolism-related changes have been reported in ageing^12,16^. The candidate genes were filtered based on entropy changes. We provide experimental evidence available from the literature for the involvement of candidate genes in ageing or its related liver diseases. Top novel candidate genes with high entropy values include VTN, FGB and FGG, which are associated with changes observed in fibrosis under chronic liver damage condition^38^. We hypothesize that transcriptional remodelling of the liver during ageing can affect the integrity of the membrane and increase the susceptibility to fibrosis. Ageing is shown to increase the susceptibility to fibrosis in response to high-fat diet feeding^39^. We also found genes of the complement system (C6, C8, C8A, C8B) that are part of the membrane attack complex changing with ageing (Fig. 3B). There is increasing evidence that complement systems may play a role in ageing^40^.

Our analysis also revealed the PGRMC1-INSIGs-SREBF1 axis in controlling the lipid levels in ageing (Fig. 3B). PGRMC1 knockout leads to the buildup of fatty acids and predisposes mice to NAFLD^41^. PGRMC1 forms complex with INSIG1 and is associated with cleavage of SREBF1 via SCAP^41,42^. Deletion of INSIG2 also results in the activation of SREBF1 and de novo lipid synthesis^43^. Age induced hepatic steatosis is alleviated in INSIG2 elevated condition^44^. Another candidate gene, PLIN2, also controls the activation of SREBP-1 and SREBP-2^45^. Its expression is shown to be altered in age-related diseases, including fatty liver^46,47^. Fat accumulation is negatively correlated with the decrease in mitochondrial mass with ageing^48^. Further, ageing is shown to affect lipid homeostasis by controlling the phosphorylation of CEBPα/β^49^ and changing the nucleosome occupancy at the foci of PPAR targets^50^. We found that PPARA can also be affected through CREB3L3, the knockout of which results in severe fatty liver^51^. CEBPβ is implicated in the activation of SREBF1 transcription in liver^52^. RARRES2 (Chemerin) is another candidate ageing gene, which is also induced in NAFLD and Hepatitis B-related HCC. These observations suggest that ageing may increase susceptibility to liver diseases.

The network topology-based analysis of the ageing network revealed the involvement of ribosomes and proteasomes, which reflects the changes in the proteostasis capacity of cells with ageing^53^. The module associated with oxidative phosphorylation in the ageing network (Fig. 4) reflects the change in mitochondrial metabolism with ageing^3^. We found Wnt pathway as an ageing module, which controls cell renewal, tissue regeneration and the development of HCC^54^. Further, TRP53 was identified as a critical node based on local and global graph theoretical measures. It has relevance in ageing as it can promote repair, survival, or elimination of damaged cells^55^. TRP53 optimally balances tumor suppression and longevity^56^. The decline in the function of TRP53 is observed in various tissues of the mouse with ageing, which can contribute to increased mutation frequency and tumorigenesis^57^. Other critical nodes include AKT1, SRC, CTNNB1 and EGFR, which are related to cancer signalling. CTNNB1 encodes a β-catenin protein responsible for controlling gene expression in the Wnt signalling pathway. EGFR also shows an increase in entropy, and its expression is correlated with liver steatosis in mice and human^58^.

The PPI network analysis of ageing and different liver conditions also shows the proximity of ageing genes to different liver conditions, including NAFLD, HCC, liver damage and repair (Fig. 5). The common theme shared between conditions maps to immune-related pathways, pathways in cancer and metabolic changes. MAPK, PI3K-AKT, Ras, Wnt and NFκB signalling are common pathways across conditions (Fig. 6C). Studies on extended lifespan by pharmacological intervention suggested that anti-ageing effects are mediated by targeting the canonical MAPK pathway^59^. With ageing, there is an upregulation of MEK1, which triggers translation by phosphorylating its downstream target eIF4E^59^. Increased activity of eIF4E has been shown to promote tumorigenesis, thus implicating ageing effects on cancer^60^. GSK3β is a common node across conditions. Ageing is shown to inhibit GSK3β function^61^ and this, in turn, affects the liver regeneration potential^62^. Inhibition of GSK3β acts as a protective role against lipid accumulation in NAFLD. GSK3β can regulate cell proliferation by controlling the growth-inhibitory activity of CEBPα and negatively regulates many oncogenic signalling pathways, such as the Wnt/β-catenin pathway^63^. We found GSK3B-CTNBB1 interaction as part of the common theme (Fig. 7), which is linked to HCC development and NAFLD. There is also a mechanistic link between inflammation and the development of HCC mediated by NFKβ signalling^64^. NASH condition exhibits morphological conditions related to infiltration of lymphocytes and neutrophils, hepatocyte death and activation of liver resident macrophages Kupffer cells, creating an environment favourable for compensatory hepatocyte proliferation that further drives hepatocarcinogenesis^65^. Further, the priming phase of liver regeneration after PH depends on the activation of NFKβ^66^.

We observed lipid metabolism as a common theme across ageing and liver-associated conditions. Induced alteration in lipid metabolic genes in ageing may increase susceptibility to NAFLD and affect liver regeneration. Both lipid overloading and deficiency can affect the liver regeneration ability. Fine-tuning lipid levels by transport, biosynthesis and oxidation is crucial for liver regeneration^67^. A high fat diet impairs liver regeneration through IKKβ overexpression and subsequent NFKβ inhibition^68^. The aberrant activation of FASN plays a major role in the development of HCC and its level is also shown to increase during the induction of senescence^69^.

In amino acid metabolism, one-carbon metabolism is altered across conditions, and it plays a crucial role in maintaining tissue homeostasis and longevity^70,71^. It generates various metabolites that are building blocks of nucleotide synthesis, methylation, and redox reactions. Oncogenic signalling hijacks the one-carbon metabolism to support proliferation and survival^72^. Genetic disruption of MAT1 inhibits liver regeneration^73^. MAT1 expression is reduced in different liver pathologies, including NAFLD and HCC. Hepatic methionine is depleted in mice that developed NAFLD, and administration of methionine and choline-deficient diet led to alterations in the expression of lipid metabolism genes^74,75^. Metabolomics analysis of ageing shows the levels of serine and methionine decrease in liver^76^. These highlight the importance of one-carbon metabolism in liver function and pathology. Further, BCAA is altered across conditions, and loss of BCAA catabolism promotes HCC development and progression^77^. However, this is not suppressed in liver regeneration^73^. BCAA metabolites are also altered in aged liver^3^.

In summary, our study maps the network-level changes of ageing and dissects the crosstalk between different conditions, including regeneration and diseases in the mouse liver. We uncovered the local and global changes in immune response, cancer signalling and metabolism with ageing and identified novel candidate genes. We showed the proximity of the liver ageing network to liver-condition-specific networks and identified the interconnections through common pathways. This explains how ageing increases susceptibility to different disease conditions and affects the capacity of the liver to regenerate.

As an initial study, we used the bulk sequencing data to generate a liver tissue-specific PPI network in different contexts for comparison. The bulk changes can be due to cell composition changes or alterations in the gene expression of each cell in the population. The single-cell data will further help to refine the interactions in a cell-type specific manner. Nevertheless, our study provides the initial framework for single-cell network analysis of liver ageing and its related diseases. We will also extend our analysis pipeline to human liver aging, transplantation, and associated pathologies.

## Supplementary Information

Supplementary Information.

## Data Availability

All the datasets are freely available and can be downloaded from https://www.ncbi.nlm.nih.gov/geo/ using the given accession numbers in Table 1.
