# Supplementary material for: Network-level analysis of ageing and its relationship with diseases and tissue regeneration in the mouse liver
Source: Sci Rep. 2023 Mar 21;13:4632. doi: 10.1038/s41598-023-31315-2 (PMC10030664; doi:10.1038/s41598-023-31315-2)
Supplement: Supplementary file 1 — Supplementary Information. [file 41598_2023_31315_MOESM1_ESM.pdf]

Supplementary information

Network-level analysis of ageing and its relationship with diseases and tissue regeneration in the mouse liver

Manisri Porukala and P K Vinod*

Centre for Computational Natural Sciences and Bioinformatics, IIIT, Hyderabad-500032, India

*Correspondence: vinod.pk@iiit.ac.in

Phone: +91 40-66531177

Supplementary methods

Network entropy:

The PPI network with N nodes forming an adjacency matrix $A = \{a_{ij}\}\ \forall\ i, j \in 1,2,....,N$ with edge weights d_{ij} has two sets of observables related to topology and distribution of edge weights. Topology is an N-dimensional vector $\{k_i\}\ \forall\ i \in 1,2,....,N,$ called degree sequence with the number of connections of each node. The distribution of edge weights is related to partitioning distance between nodes into N_b bins, where $N_b = \sqrt{N}$. The edge weight between nodes i and j of sample a is given as the Euclidean distance of the gene expression (g) values i.e.,

$$d_{ij} = \sqrt{\left(g_i^a - g_j^a\right)^2} = \left|g_i^a - g_j^a\right|$$

Each bin takes a value corresponding to the number of links whose associated edge weights fall inside its boundaries. A network ensemble is imposed to have a topological constraint similar to the degree sequence of the sample-specific network and the spatial constraint to have the same average value of links per bin. Therefore, topological and spatial constraints based on the expression profile are specific for each sample. The entropy of the network ensemble is defined as

$$S = -\sum_{i<j} p_{ij} \log p_{ij} - \sum_{i<j} \left(1 - p_{ij}\right) \log\left(1 - p_{ij}\right)$$

where p_{ij} is the probability of finding a link between node i and node j. As with the canonical entropy, the members of an ensemble on an average satisfy the constraints.

The constraints on the degree sequence k_i and link distribution B_l are defined as follows:

$$k_i = \sum_{j}^{N} p_{ij}\ ;\ \ i = 1,\ldots,N$$

$$B_l = \sum_{i<j}^{N} \chi_l\left(d_{ij}\right) p_{ij};\ \ l = 1,\ldots,N_b$$

χ_l is the characteristic function of each bin of width $(\Delta d)_l$ such that $(\Delta d)_l : \chi_l(x) = 1\ if\ x \in [d_l\ , d_l + (\Delta d)_l]$, $\chi_l = 0\ otherwise$

The probability matrix $\{p_{ij}\}$ is obtained by constrained maximisation of entropy function as:

$$\frac{\partial}{\partial p_{ij}}\left\{S + \sum_{i}^{N}\lambda_i\left(k_i - \sum_{j} p_{ij}\right) + \sum_{l}^{N_b} g_l\left(B_l - \sum_{i<j}^{N}\chi_l(d_{ij})p_{ij}\right)\right\} = 0$$

where λ_i and g_l are the Lagrangian multipliers related to the constraints. For each pair of i and j, the marginal probability is given as

$$p_{ij} = \sum_{l}^{N_b}\chi_l(d_{ij})\frac{e^{-(\lambda_i+\lambda_j+g_l)}}{1 + e^{-(\lambda_i+\lambda_j+g_l)}} = \sum_{l}^{N_b}\chi_l(d_{ij})\frac{z_i z_j W_l}{1 + z_i z_j W_l}$$

where $z_i = e^{-\lambda_i}$ and $W_l = e^{-g_l}$ are functions of the Lagrangian multipliers λ_i and g_l.

The link probabilities p_{ij} obtained from the full PPI network of a sample can be further used to derive the entropy associated with a single node i that takes the form of Shannon entropy. This is valid since $p_{ij} > 0,\ \forall\, i = 1,2,....,N$ and $\sum_j p_{ij} = k_i$, the degree of node i. Single-node entropy S_i of *i-th* node is given as:

$$S_i = -\sum_{j} p'_{ij}\ \log p'_{ij}\,; \quad p'_{ij} = \frac{p_{ij}}{k_i}$$

Table S1: Pathway enrichment of top 3 MCODE clusters from the PPI network obtained using the DEGs between 3 and 18 month old mice and their first neighbours.

Cluster	Nodes	Edges	Score	KEGG Pathway	Overlap	adj p-value
1	52	1240	48.627	Oxidative phosphorylation	45/134	3.66e-93
				Parkinson disease	45/144	8.99e-92
				Non-alcoholic fatty liver disease (NAFLD)	45/151	7.57e-91
				Alzheimer disease	45/175	1.30e-87
				Huntington disease	45/192	1.23e-85
2	55	710	26.296	Cell cycle	8/123	2.80e-08
				Progesterone-mediated oocyte maturation	7/90	4.52e-08
				Oocyte meiosis	6/116	4.61e-06
				p53 signaling pathway	3/71	4.43e-03
				Cellular senescence	4/185	6.02e-03
3	28	342	25.333	Ribosome	17/170	5.5e-29

Table S2: Pathway enrichment of top 5 MCODE clusters from the PPI network obtained using the DEGs between 3 and 24 month old mice and their first neighbours.

Cluster	Nodes	Edges	Score	KEGG Pathway	Overlap	adj p-value
1	87	2875	66.860	Proteasome	40/46	8.08e-91
				Epstein-Barr virus infection	28/229	1.31e-31
				Basal cell carcinoma	5/63	2.83e-04
				Human T-cell leukemia virus 1 infection	8/245	2.83e-04
				p53 signaling pathway	5/71	2.83e-04
2	32	481	31.032	Thyroid hormone signaling pathway	11/115	1.62e-17
3	52	572	22.431	DNA replication	13/35	2.33e-24
				Cell cycle	16/123	2.23e-22
				Nucleotide excision repair	6/43	1.47e-08
				Ubiquitin mediated proteolysis	6/138	1.29e-05
				Circadian rhythm	3/30	4.35e-04
4	62	632	20.721	Collecting duct acid secretion	16/27	5.24e-33
				Synaptic vesicle cycle	18/77	1.54e-28
				Rheumatoid arthritis	18/84	5.89e-28
				Oxidative phosphorylation	18/134	3.84e-24
				Phagosome	18/180	7.76e-22
5	17	135	16.875	Peroxisome	12/84	1.13e-24

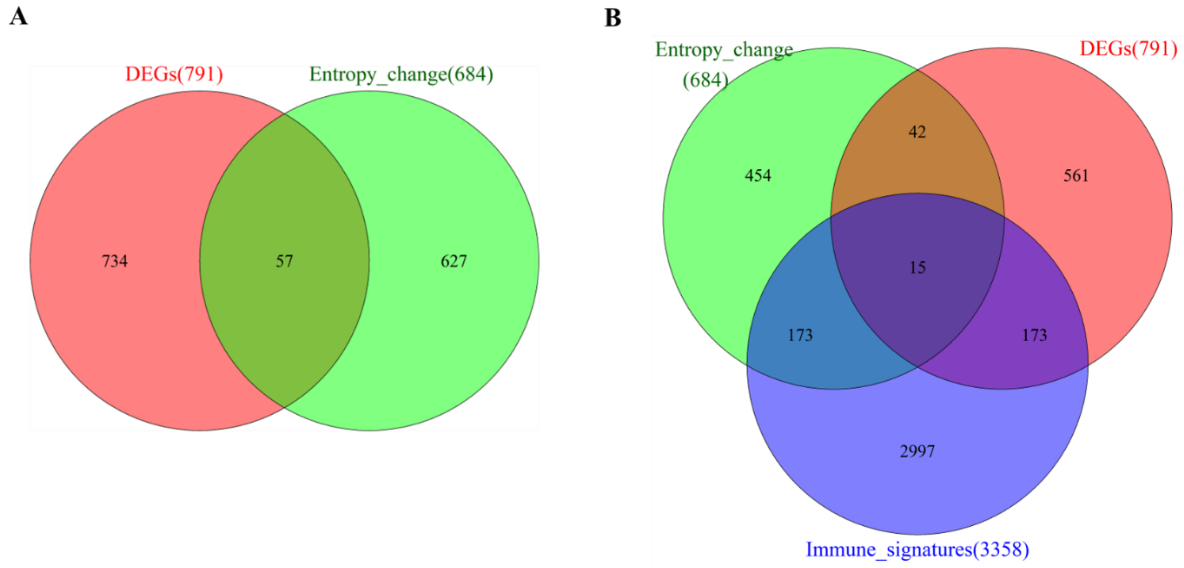

Figure S1: Venn diagrams showing the overlap of entropy-based candidates, ageing DEGs and immune signatures.

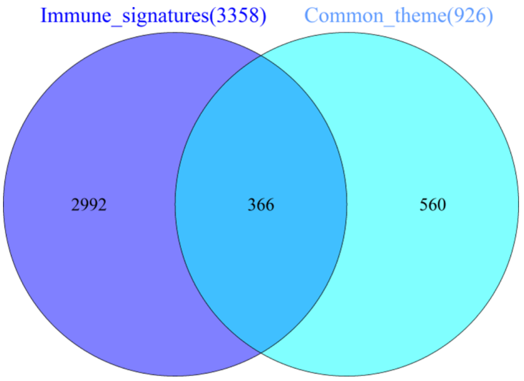

Figure S2: Venn diagram showing the immune signatures in common theme shared by different pathological conditions
